# An assessment of the immune status of some stone quarry workers in Ondo state, Nigeria

**DOI:** 10.1097/MD.0000000000036969

**Published:** 2024-01-12

**Authors:** Samson O. Onemu, Emmanuel Ifeanyi Obeagu, Adeniyi Adewumi Popoola, Michael A. Osuntuyi, Clement N. Isibor

**Affiliations:** a Department of Medical Laboratory Science, Achievers University, Owo, Nigeria; b Department of Medical Laboratory Science, Kampala International University, Kampala, Uganda; c Department of Geological Sciences, Achievers University, Owo, Nigeria; d Department of Biological Sciences, University of Delta, Agbor, Nigeria.

**Keywords:** adverse effects, immune status, inhalation, silica dust, stone quarrying

## Abstract

Stone quarry activities in Nigeria are mostly unregulated such that the workers in these quarries are continuously exposed to the inhalation of silica dust. It has been observed that silica dust particles negatively impact the health of stone quarry workers which usually manifest as respiratory difficulties, asthma-like illnesses and other adventitious events of the lungs. The study was designed to evaluate the probable immunological impact of silica dust inhalation from stone crushing by workers. Blood samples were collected from consenting workers and analyzed for total white blood cells and their subsets. Absolute CD4 cells numbers were also determined. The results indicated that neutrophils and eosinophils numbers increased significantly (*P* < .05) and CD4 counts declined significantly (*P* < .001). Alteration in these proportions is a pointer to the injurious impact of silica dust on the immune system of these workers. The findings in this study should spur actions in the education of these workers on the need for the use of proper personal protection equipment and the establishment of a scheme to periodically carry out a health assessment check to identity those at most risk of developing chronic illnesses.

## 1. Introduction

Quarrying stones contain as much as 77% silica that are released in dust particles during crushing. Continuous inhalation silica dust overtime has been linked with silicosis, lung neoplasms, severe respiratory difficulties, impaired oxidative activities, and depressed immune responses,^[1]^ including a specific form of a fibrous lung.^[2]^ Stone quarrying processes are carried out in Nigeria without a definite regulatory framework to protect the workers and the environment.^[3]^ In India, where there is also no specific regulation, the highest rate of occupational associated harm has been documented in workers.^[4]^ Inhalation of dust from mining activities and those living in close proximity to mining sites have been shown to be prone to respiratory illnesses and lung tumors.^[6,7]^ Stone quarry activities just like other mining processes generally involve the liberation of inhalable particles of different sizes that are readily taken-in with air by the workers and those living in the immediate neighborhood. This has been shown to compromise the health of the workers in a variety of ways that include the development of asthma, lung tumors and pneumoconiosis,^[8]^ and a decline in respiratory health.^[9]^ Silica particles are known to facilitate oxidative stress as a mechanism for toxicity.^[10]^ Quarry stones are sourced for building and construction purposes, but they often possess radionuclides of uranium, thorium and potassium that pose some high degree of radioactivity.^[11–13]^ The danger posed by metalliferous dust does not depend only on their superfluity and fine particle sizes but also on their chemical interaction with tissues and organs of those who inhale these particles.^[14]^ This is often accompanied with various health consequences.^[15]^ Dust from mining environments have traditionally been observed to be associated with widespread fatal illnesses that range from pneumoconiosis originating from silicosis or the usual coal workers pneumoconiosis or black lung arising from inflammatory response in the lungs^[16]^ and the consequential decline in the function of the lungs.^[17]^ The lung is remarkably affected by quarry stone dusts that are inhalable and possess the capacity to initiate congestive obstruction in the lungs resulting from inflammatory reaction,^[18,19]^ and the tendency for lung related illnesses to exacerbate with the passage of time.^[20]^ Studies in Nigeria and from other places have shown significantly raised counts in white blood cells’ (WBCs) populations of stone quarry workers.^[21,22]^ Elevated eosinophils have been specifically documented as signal for deteriorating health of stone miners.^[23]^ The present study was conceived to evaluate the immunological impact of stone quarry dust on the health of workers in Ondo state, Nigeria.

## 2. Materials and methods

### 2.1. Study design

A case–control study was adopted for the research.

### 2.2. Study area

The study was conducted in Akure Local Government Area Council, Akure, Ondo state.

#### 2.2.1. Population.

The population consisted of workers at the stone quarry at Akure Local Government Area Council, Akure, Ondo state. Workers who gave informed consent and have been working in the quarry for a minimum of 6 months prior to the study period were recruited.

### 2.3. Laboratory investigation

Each sample was analyzed for total WBCs, using Mythic-18 Hematology Analyzer (Model 2022). Absolute CD4 numbers was determined with PARTEC^®^ Cyflow Counter-2.

### 2.4. Ethical approval and consent

A written permission approved by the management of the quarry was obtained and ethical approval was granted by Ondo State Ministry of Health Ref. No. OSHREC.14/03/23/521 dated March 14, 2023. Written consents were obtained from the subjects before commencement of the study.

#### 2.4.1. Sample size.

The sample size was calculated with formula


$$\begin{array}{l} n=z^{2}\left(p-q\right)/d^{2} \end{array}$$

n = desired sample size.

z = standard normal deviate that corresponds to 95% confidence limit.

P = prevalence rate or 23.1% quarry worker in Ondo state region.

q = 1−p.

∴n = (1.94)^2^ × 0.231 (1 − 023.1)/00.5^2^

= (1.96) × 0.231(1−0.231)/0.0025 = 147.6.

A minimum of 148 samples will be required.

### 2.5. Sample and data collection

Biographical data was collected from each subject. This was followed with the collection of 5 mL of blood through venipuncture and dispensed into an EDTA bottle, well mixed, labeled and placed in a cold chamber for transportation to the laboratory. Samples were also collected from apparently healthy, age-matched volunteer non-quarry workers who do not reside in close proximity to a quarry as control.

### 2.6. Data analysis

Data analysis was done with the aid of Statistical Package for Social Sciences version 20 (SPSS v. 20). The *t*-test was used to compare white blood cells and their different subsets between quarry workers and the control group.

## 3. Results

A total of 148 samples were collected from stone quarry workers and 124 non-quarry workers. The mean age of the quarry workers was 39.35 years (mean ± SD; 39.35 ± 6.99) and only men were involved in stone crushing at this quarry. The few women involved had task of gathering and transporting the crushed stones. The total while blood cell and their sub-sets are shown in Table 1. The total WBCs in the quarry workers were generally higher but the difference was not significant statistically (*P* > .05). The values for basophils and monocytes were not also significantly different (*P* > .05). The lymphocytes population heightened significantly. (*P* < .05) in comparison to the control group. Eosinophils numbers were also significantly elevated (*P* < .05) in quarry workers. Absolute CD4 numbers were significantly depressed (*P* < .001) in the quarry workers.

**Table 1:** Mean white blood cells (WBCs) counts and their subsets.

Parameter	Quarry worker	Non-quarry worker	*P*-value
Total WBCs (×10^3^/µL)	7.30 ± 6.30	6.50 ± 2.22	.246
Neutrophils (%)	39.10 ± 11.20	53.30 ± 3.47	.049
Lymphocytes (%)	52.80 ± 8.22	32.25 ± 5.50	.007
Eosinophils (%)	4.37 ± 0.98	0.72 ± 028	.004
Basophils (%)	0.87 ± 0.98	0. 72 ± 1.55	.176
Monocytes (%)	0.95 ± 0.98	0.99 ± 0.96	.552
AbsoluteCD4 cells/µL	410 ± 119.19	789.4 ± 25.03	<.001

## 4. Discussion

Stone quarry activities in Akure involved mainly men as opposed to both sexes in Abakaliki, South-Eastern Nigeria.^[24]^ Workers in stone quarrying sites in Akure share a common denominator of being lowly educated, and who had no trade skills with a mean age of 39.35 ± .99 years. The analysis of blood samples from the workers demonstrated that some WBCs populations were altered. The total WBCs were elevated in the quarry workers but, this was not at a significant level as other studies have indicated.^[21,24]^ Neutrophils counts increased significantly (*P* < .05), and neutrophils being a subset of WBCs, a rise in their numbers is an inference of a challenge to immune status of the study subjects as other studies have indicated.^[20,21,24]^ This may also suggest that elevated neutrophils numbers point to a functional challenge of the host defense mechanism as may be exemplified by their role in cancer cells and during the use of cytotoxic agents that results in neutropenia and the resultant high fatality.^[25,26]^ Lymphocytes counts in the study subjects were heightened significantly (*P* < .05) in comparison to the control group. Lymphocytes are the major cells involved in adaptive immunity, specializing as helper type of cells (Helper T-Cells or T_H_-Cells) which in coordinated manner assist in the elimination of invading microorganisms. It is obvious to envisage that the effect of silica dust on the respiratory systems increases the vulnerability of the workers to a variety infection of opportunities especially those of respiratory tract and other lung diseases or cancer.^[5]^ Significantly elevated levels of eosinophils (*P* < .05) were also recorded. This has been observed in prior studies.^[27,28]^ Raised numbers of eosinophils are often associated with infectious disorders and for some varieties of cancers.^[29]^ Eosinophils may therefore serve as signal of a waning immunity in the study population. Absolute CD4 cells counts declined significantly (*P* < .001) in the study population. These specialized cells which are a subgroup of T-lymphocytes and important component of the host defense against infectious agents and wholesomeness, a decline in their absolute numbers definitely raises an alarm to the possible development of a disease process. This finding has simulated reports from other studies.^[30,31]^

## 5. Conclusion

Elevated neutrophils, eosinophils and depressed CD4 counts are important markers of compromised immunity in stone quarry workers. Stone quarry activities in Ondo state expose the workers to the hazard of continuous inhalation of silica dust that represent a significant source of a health challenge that is eagerly waiting to implode. Education and use of proper personal protection equipment will be a first step towards intervention and mitigation. It is also necessary to periodically carry out a health assessment check on these workers to forestall the development chronic illnesses associated with mining activities.

## Author contributions

**Conceptualization:** Samson O. Onemu.

**Data curation:** Samson O. Onemu, Michael A. Osuntuyi.

**Investigation:** Samson O. Onemu, Michael A. Osuntuyi.

**Methodology:** Samson O. Onemu, Emmanuel Ifeanyi Obeagu, Michael A. Osuntuyi, Clement N. Isibor.

**Supervision:** Samson O. Onemu.

**Validation:** Emmanuel Ifeanyi Obeagu.

**Visualization:** Emmanuel Ifeanyi Obeagu, Adeniyi Adewumi Popoola.

**Writing – original draft:** Samson O. Onemu, Emmanuel Ifeanyi Obeagu, Adeniyi Adewumi Popoola, Michael A. Osuntuyi, Clement N. Isibor.

**Writing – review & editing:** Emmanuel Ifeanyi Obeagu, Adeniyi Adewumi Popoola, Clement N. Isibor.
